# Multimodal neuromonitoring-guided cerebral protection and early neurocognitive outcomes after aortic arch surgery

**DOI:** 10.3389/fphys.2026.1948101

**Published:** 2026-09-22

**Authors:** Xue Zhang, Xuan Jing, Ruixia Hao, Na Xu, Chunmin Zhang, Dong Wang, Wei Liu, Pengting Liu

**Affiliations:** 1 Shanxi Bethune Hospital, Shanxi Academy of Medical Sciences, Third Hospital of Shanxi Medical University; Tongji Shanxi Hospital, Nursing Department, Taiyuan, China; 2 Shanxi Bethune Hospital, Shanxi Academy of Medical Sciences, Third Hospital of Shanxi Medical University; Tongji Shanxi Hospital, Department of Anesthesiology, Taiyuan, China; 3 Shanxi Bethune Hospital, Shanxi Academy of Medical Sciences, Third Hospital of Shanxi Medical University; Tongji Shanxi Hospital, Department of Cardiovascular Surgery, Taiyuan, China

**Keywords:** aortic arch surgery, cerebral autoregulation, cognitive decline, multimodal neuromonitoring, near-infrared spectroscopy, postoperative delirium, processed electroencephalography, selective antegrade cerebral perfusion

## Abstract

**Background:**

Open aortic arch surgery exposes patients to hypothermic circulatory arrest, selective antegrade cerebral perfusion, rewarming, and arch-vessel manipulation. Postoperative delirium and early cognitive decline remain clinically important complications. Because single-modality cerebral oximetry cannot fully capture perioperative cerebral physiological burden, this study evaluated whether a multimodal neuromonitoring-guided cerebral-protection protocol was associated with lower cerebral physiological burden and improved early neurocognitive outcomes after aortic arch surgery.

**Methods:**

This retrospective single-center cohort study screened consecutive adult patients who underwent open aortic arch surgery from January 1, 2021 to April 30, 2026. Patients treated from January 1, 2021 to December 31, 2023 formed the conventional cerebral-protection cohort, and patients treated from January 1, 2024 to April 30, 2026 formed the multimodal-guided cohort. The guided protocol used bilateral near-infrared spectroscopy, processed electroencephalography, transcranial Doppler, and cerebral oximetry index-derived autoregulation monitoring to trigger correction of perfusion pressure, selective antegrade cerebral perfusion flow, cannula position, PaCO_2_, hemoglobin, anesthetic depth, and rewarming speed. The primary outcome was postoperative delirium within postoperative days 1–5. The main secondary cognitive outcome was MoCA-defined 30-day cognitive decline. Inverse probability weighting and multivariable regression were used to address baseline imbalance and calendar-period confounding. A patient-level segmented time-series model was also used to estimate the pre-implementation temporal slope, the immediate level change at protocol implementation, and the post-implementation slope change.

**Results:**

The final cohort included 186 patients, with 92 in the conventional cohort and 94 in the multimodal-guided cohort. The guided cohort had lower absolute rSO_2_ burden, shorter EEG suppression burden, and shorter duration with impaired cerebral autoregulation. Postoperative delirium occurred in 36 of 92 patients in the conventional cohort and 14 of 94 patients in the guided cohort. MoCA-defined 30-day cognitive decline occurred in 31 of 83 patients with available follow-up in the conventional cohort and 10 of 90 patients in the guided cohort. In weighted adjusted models, the guided cohort was associated with lower odds of postoperative delirium (OR, 0.36; 95% CI, 0.17–0.77) and lower odds of 30-day cognitive decline (OR, 0.31; 95% CI, 0.13–0.75). The delirium association was attenuated after adding intraoperative cerebral physiological burden variables. In segmented analyses, neither outcome showed a significant pre-implementation trend; protocol implementation was associated with an immediate reduction in the odds of postoperative delirium (level-change OR, 0.20; 95% CI, 0.05–0.91) and 30-day cognitive decline (OR, 0.17; 95% CI, 0.03–0.92), without evidence of an additional post-implementation slope change.

**Conclusions:**

In adult aortic arch surgery, implementation of a multimodal neuromonitoring-guided cerebral-protection protocol was associated with lower intraoperative cerebral physiological burden, lower postoperative delirium risk, and less MoCA-defined 30-day cognitive decline. These findings support the use of complementary neuromonitoring signals within a structured correction pathway rather than reliance on a single cerebral oxygenation threshold.

## 1 Introduction

Open aortic arch surgery remains one of the most neurologically demanding procedures in cardiovascular surgery because arch repair often requires cardiopulmonary bypass, hypothermic circulatory arrest, selective antegrade cerebral perfusion, arch-vessel manipulation, and controlled rewarming. Although contemporary cerebral-protection strategies have reduced catastrophic neurological injury, stroke, transient neurological dysfunction, delirium, and early cognitive decline still occur after complex arch repair (Benedetto et al., 2022). These complications are unlikely to arise from a single mechanism. During the same operation, the brain may be exposed to hypoperfusion, embolic load, interhemispheric flow mismatch, anemia, altered carbon dioxide tension, impaired pressure autoregulation, excessive cortical suppression, systemic inflammation, and reperfusion stress (Trivedi et al., 2024).

Selective antegrade cerebral perfusion is widely used to maintain cerebral blood flow during circulatory arrest, but adequate perfusion cannot be inferred from pump flow or cannula position alone. Middle cerebral artery velocity, frontal regional cerebral oxygen saturation, cerebral perfusion pressure, and bilateral oxygenation symmetry may change differently during cooling, circulatory arrest, selective perfusion, rewarming, and separation from cardiopulmonary bypass (Olsson and Thelin, 2006). Earlier intraoperative studies using transcranial Doppler and near-infrared spectroscopy during antegrade selective cerebral perfusion showed that these techniques measure complementary aspects of cerebral perfusion and oxygenation during arch surgery (Yu et al., 2001). NIRS-based protocols have also been used to adjust bilateral antegrade cerebral perfusion and detect hemispheric desaturation during aortic arch repair (Urbanski et al., 2010).

Postoperative delirium is a clinically important neurological outcome after cardiac surgery. It is associated with older age, lower preoperative cognitive reserve, emergency surgery, comorbidity burden, intraoperative physiological stress, longer intensive-care stay, and delayed functional recovery (Feng et al., 2024). In aortic arch surgery, delirium is especially relevant because patients are exposed to circulatory arrest, selective cerebral perfusion, major vascular manipulation, temperature shifts, and high postoperative inflammatory burden. Delirium is also not limited to the immediate ICU period; cardiac-surgery studies have shown that patients who develop delirium may have worse early postoperative cognitive trajectories, including greater cognitive decline at approximately 1 month after surgery (Brown et al., 2018).

Near-infrared spectroscopy is a practical tool for detecting cerebral oxygen desaturation during cardiac surgery, but it should not be treated as a complete cerebral-protection strategy. Some studies have linked cerebral desaturation to postoperative cognitive dysfunction, whereas larger analyses have not consistently shown an association between intraoperative rSO_2_ desaturation and postoperative delirium (Law et al., 2025). This inconsistency is biologically plausible because frontal oxygen saturation does not fully represent large-vessel cerebral flow, embolic events, cortical electrical suppression, pressure autoregulation, or perioperative inflammation.

Processed electroencephalography, transcranial Doppler, and cerebral autoregulation monitoring therefore provide important complementary information. EEG suppression during cardiac surgery has been associated with postoperative delirium, supporting the role of cortical electrical vulnerability and anesthetic-metabolic balance in postoperative brain dysfunction (Soehle et al., 2015). NIRS-derived cerebral oximetry index can identify pressure-passive cerebral oxygenation during cardiopulmonary bypass and may help individualize mean arterial pressure targets when cerebral autoregulation is impaired (Brady et al., 2010). TCD can further identify cerebral flow asymmetry or abrupt flow loss that may not be visible from NIRS alone, particularly during selective antegrade cerebral perfusion and arch-vessel manipulation.

The relevant clinical question is not whether one monitoring device can independently prevent delirium, but whether a structured multimodal monitoring protocol can reduce measurable cerebral pathophysiological burden and improve early neurocognitive outcomes. This study evaluated a time-defined institutional transition from conventional cerebral protection to a multimodal neuromonitoring-guided cerebral-protection protocol in adult open aortic arch surgery. We hypothesized that the guided protocol would be associated with lower intraoperative cerebral oxygenation burden, less EEG suppression burden, shorter impaired autoregulation duration, lower postoperative delirium risk, and less MoCA-defined 30-day cognitive decline.

## 2 Methods

### 2.1 Study design and data source

This retrospective single-center cohort study was conducted at Shanxi Bethune Hospital. Consecutive adult patients who underwent open aortic arch surgery from January 1, 2021 to April 30, 2026 were screened. The 30-day follow-up window for the last eligible operation ended on June 6, 2026. Data extraction was completed from May 15 to June 10, 2026, and the database was locked on June 15, 2026. The present study was conducted under ethics approval No. YXLL-2023-029 granted by the institutional ethics committee of Shanxi Bethune Hospital. Written informed consent was waived because the study used existing perioperative records and routine follow-up data. The study followed the STROBE recommendations for observational cohort studies (von Elm et al., 2007).

The institutional cerebral-protection protocol for aortic arch surgery was revised on January 1, 2024. Patients treated from January 1, 2021 to December 31, 2023 formed the conventional cerebral-protection cohort, and patients treated from January 1, 2024 to April 30, 2026 formed the multimodal neuromonitoring-guided cerebral-protection cohort. Cohort assignment was based only on operation date relative to the institutional protocol change. During the study period, the ICU delirium-screening policy, postoperative sedation target, and 30-day cognitive follow-up pathway were not intentionally altered as part of the neuromonitoring implementation. Because cohort assignment was determined entirely by operation date, these statements do not exclude contemporaneous changes in accumulated team experience or other perioperative practices around the implementation boundary. Cohort construction, data sources, signal processing, and outcome assessment are shown in **Supplementary Figures 1A–D**.

During the study period, four attending aortic surgeons, six cardiac anesthesiologists, and five perfusionists contributed to arch cases; three surgeons, four anesthesiologists, and four perfusionists worked in both protocol periods. The center screened 43, 45, 46, 47, and 48 arch cases in 2021–2025, respectively, with 18 additional cases from January through April 2026. Among the primary arch surgeons, the median annual operator volume during complete calendar years was 11 cases (interquartile range, 9–14), and no abrupt change in operator composition or center case volume occurred at the January 2024 implementation boundary. Team continuity, center case volume, the per-surgeon annual volume summary, and major co-interventions by protocol period are summarized in **Supplementary Table 3**.

### 2.2 Eligibility criteria

Patients were eligible if they were 18–80 years old and underwent open aortic arch surgery requiring cardiopulmonary bypass, hypothermic circulatory arrest, and selective antegrade cerebral perfusion. Eligible operations included total arch replacement, partial arch replacement, frozen elephant trunk implantation, and arch repair for acute type A aortic dissection, chronic aortic dissection, or aortic arch aneurysm.

Exclusion criteria were age >80 years, preoperative coma, documented dementia, severe aphasia, inability to complete delirium or cognitive assessment in Chinese, preoperative cardiopulmonary resuscitation, active central nervous system infection, chronic dialysis, severe hepatic failure, severe carotid or intracranial stenosis requiring separate intervention before aortic surgery, death within 24 h after surgery, incomplete anesthesia record, missing cardiopulmonary bypass record, unavailable postoperative day 1–5 delirium documentation, or repeated aortic arch operation during the same admission. Patients without 30-day cognitive follow-up were retained for the primary delirium analysis, and their 30-day cognitive-change outcome was coded as missing.

### 2.3 Perioperative management and cerebral protection

Anesthesia followed the institutional cardiac anesthesia protocol. Standard monitoring included radial and femoral arterial pressure, central venous pressure, five-lead electrocardiography, pulse oximetry, nasopharyngeal temperature, bladder or rectal temperature, urine output, activated clotting time, arterial blood gas analysis, and transesophageal echocardiography. Anesthesia was induced with etomidate or propofol, sufentanil, and rocuronium. Maintenance used propofol or sevoflurane with opioid infusion. Benzodiazepines were avoided unless clinically required. Before cardiopulmonary bypass, ventilation targeted SpO_2_ ≥95%, PaCO_2_ 35–45 mmHg, and arterial pH 7.35–7.45.

Cardiopulmonary bypass was performed with a full-flow target of 2.2–2.6 L/min/m² before circulatory arrest. Mean arterial pressure was generally maintained at 50–80 mmHg during cooling and rewarming. Hematocrit was maintained at ≥24% during cardiopulmonary bypass unless bleeding or hemodilution required temporary deviation. The target nasopharyngeal temperature before circulatory arrest was 24–28 °C. Selective antegrade cerebral perfusion was delivered through right axillary artery cannulation, innominate artery cannulation, direct arch-vessel cannulation, or combined cannulation according to operative anatomy. Initial selective antegrade cerebral perfusion flow was 6–10 mL/kg/min, and cerebral perfusion pressure was maintained at 40–70 mmHg. Unilateral cerebral perfusion was converted to bilateral cerebral perfusion when persistent hemispheric desaturation, poor arch-vessel backflow, abnormal cerebral perfusion pressure, or transcranial Doppler asymmetry suggested inadequate cross-perfusion. Rewarming targeted a blood–core temperature gradient <10 °C whenever feasible. These targets were consistent with contemporary cerebral-protection principles for complex aortic arch surgery (Czerny et al., 2019). Difficult separation from cardiopulmonary bypass was defined from the anesthesia/perfusion record as requiring more than one formal weaning attempt, sustained high-dose vasoactive support, or temporary mechanical circulatory support to achieve stable separation.

Major perioperative co-interventions were similar across the two protocol periods. Propofol was the principal maintenance anesthetic in 52/92 (56.5%) conventional-period cases and 55/94 (58.5%) guided-period cases, with sevoflurane used in the remaining cases. Postoperative dexmedetomidine exposure was 30/92 (32.6%) versus 34/94 (36.2%), benzodiazepine exposure was 10/92 (10.9%) versus 8/94 (8.5%), and median 24-hour intravenous morphine-equivalent opioid exposure was 19.2 (12.6–27.8) versus 18.5 (11.9–27.1) mg, respectively. Early mobilization within 48 h occurred in 54/92 (58.7%) and 61/94 (64.9%) patients. No formal ABCDEF-type bundle was introduced at the 2024 boundary, and the institutional transfusion approach and hematocrit targets used during cardiopulmonary bypass remained unchanged. These period-specific data are summarized in **Supplementary Table 3**.

### 2.4 Multimodal neuromonitoring and correction protocol

Bilateral frontal near-infrared spectroscopy was recorded with the INVOS 5100C cerebral/somatic oximeter. Processed electroencephalography was recorded with the BIS VISTA monitor. Transcranial Doppler was performed with a DWL Doppler-BoxX system and a 2-MHz probe through the transtemporal window. The anesthesia information system provided time-stamped NIRS, BIS, arterial pressure, temperature, blood gas, hemoglobin, and perfusion data. TCD measurements were extracted from the intraoperative neuromonitoring sheet. TCD was intermittent rather than continuous in both periods; when TCD was performed, the recorded measurements were abstracted from the intraoperative neuromonitoring sheet. A schematic of the intraoperative arrangement of the NIRS, BIS, TCD, and anesthesia-information-system displays is provided in **Supplementary Figure 4**. The same TCD abstraction source was used in both periods: only measurements explicitly documented on the intraoperative neuromonitoring sheet were abstracted. In the conventional period, absence of a request-based TCD entry was treated as unavailable rather than as a normal finding, so the sheet was not used to infer phase-level completeness.

INVOS 5100C and BIS VISTA values were archived at 10-second intervals through the same anesthesia-information-system interface throughout both study periods; minute-level rSO_2_ and EEG burden variables were derived after artifact rejection from this archived stream. Valid NIRS coverage across baseline, cooling, selective ACP, rewarming, and end-CPB phases was 98.2%, 97.1%, 95.8%, 96.3%, and 97.5% in the conventional cohort and 98.7%, 98.1%, 97.5%, 97.8%, and 98.4% in the guided cohort, respectively. Corresponding BIS coverage was 95.0%, 94.2%, 92.6%, 93.4%, and 94.7% versus 96.8%, 96.1%, 95.2%, 95.7%, and 96.5%. TCD examinations were performed by one of three cardiac anesthesiologists who had completed institutional perioperative TCD competency assessment after at least 25 supervised examinations; because conventional-period TCD was request-based, phase-level TCD completion was not directly comparable, and overall interpretable availability is reported instead.

In the conventional cohort, cerebral protection was guided by routine hemodynamic, perfusion, temperature, blood gas, and surgical information. NIRS and BIS values were documented but were not linked to a fixed correction pathway. TCD was performed when requested by the anesthesia or perfusion team. In the guided cohort, bilateral NIRS, BIS, and scheduled TCD assessments were linked to a prespecified correction protocol. NIRS was used for regional cerebral oxygenation, BIS for cortical suppression, and TCD for middle cerebral artery flow velocity and hemispheric flow asymmetry during arch repair (Thudium et al., 2021).

Baseline rSO_2_ was defined as the stable bilateral frontal value after anesthesia induction and before skin incision under FiO_2_ 0.50, mean arterial pressure 65–80 mmHg, and PaCO_2_ 35–45 mmHg. TCD measured M1 middle cerebral artery mean flow velocity at 45–55 mm depth. Scheduled TCD time points in the guided cohort were after induction, after cardiopulmonary bypass initiation, during cooling, 5 min after selective antegrade cerebral perfusion initiation, every 15 min during circulatory arrest, after any cerebral-perfusion adjustment, during rewarming, after separation from bypass, and before transfer from the operating room. These scheduled examinations were intermittent assessments and did not constitute continuous insonation. If the transtemporal acoustic window was absent, TCD was coded as unavailable. Standardized continuous bilateral insonation for embolic monitoring was not performed; therefore, microembolic-signal counts and a decibel detection threshold were not included as study variables in the analysis. NIRS, BIS/processed EEG, COx, and TCD monitoring ended before transfer from the operating room and were not routinely continued in the ICU; postoperative neurological surveillance relied on clinical examination, RASS, and scheduled CAM-ICU/CAM assessment.

In the guided cohort, intervention was triggered by rSO_2_ <55% for ≥3 min, rSO_2_ decrease >20% from baseline for ≥3 min, left–right rSO_2_ difference >15% for ≥3 min, MCA velocity asymmetry >30%, sudden loss of ipsilateral MCA signal after cannulation or arch-vessel manipulation, BIS <20 or suppression ratio >20% at core temperature >34 °C, or cerebral oximetry index >0.30 for ≥10 min. Correction followed a stepwise sequence: sensor and head-position check, arterial-line and cannula-position check, increase in mean arterial pressure or selective antegrade cerebral perfusion pressure, increase in pump or cerebral-perfusion flow by 10–20%, FiO_2_ increase, PaCO_2_ correction toward 40–45 mmHg, hemoglobin correction when persistent desaturation occurred with hematocrit <24–26%, reduction of excessive anesthetic depth outside cooling or circulatory arrest, slowing of rewarming, and conversion from unilateral to bilateral selective antegrade cerebral perfusion when hemispheric imbalance persisted. This sequence targeted oxygen delivery, carbon dioxide tension, hemoglobin, pump flow, perfusion pressure, cannula position, and cerebral metabolic demand (Denault et al., 2007). For the episode-level correction analysis, an NIRS-trigger episode was considered successfully corrected when all active NIRS trigger criteria had cleared for at least 3 consecutive minutes within 10 minutes after the first documented corrective action.

### 2.5 Derived cerebral pathophysiological variables

Minute-level rSO_2_ values were used for oxygenation-burden calculation. For each analyzable minute t, absolute rSO_2_ burden was calculated as max{0, 55 − max[rSO_2_,left(t), rSO_2_,right(t)]}; patient-level burden was the sum across analyzable minutes and was reported in %-min. Thus, this computational burden metric quantified periods in which the higher simultaneous hemispheric saturation was below 55%, whereas the intervention trigger remained more sensitive and could be activated by desaturation in either hemisphere. Relative rSO_2_ burden was calculated as the minute-level area below 80% of the patient-specific baseline rSO_2_. TCD asymmetry was calculated as the absolute left–right MCA mean velocity difference divided by the higher value. EEG suppression burden was calculated as the cumulative duration with BIS <20 or suppression ratio >20% at core temperature >34 °C. Cerebral autoregulation was assessed using the cerebral oximetry index, calculated as the moving Pearson correlation coefficient between 10-second averaged mean arterial pressure and rSO_2_ over 5-minute windows. COx >0.30 was treated as impaired pressure autoregulation (Brady et al., 2010). The prespecified thresholds, derived variables, and correction actions are listed in **Supplementary Table 1**.

The archived analysis specification used the same simultaneous bilateral rule described above: for each analyzable minute, the higher of the two valid frontal rSO_2_ values was compared with the 55% threshold, and the minute-level deficit was summed across the operation. Separate left- and right-hemisphere burdens were not summed or substituted for this primary burden variable. The intervention trigger and the burden metric served different purposes: the trigger was sensitive to unilateral desaturation in either hemisphere, whereas the burden metric quantified sustained bilateral/global oxygenation deficit. Isolated unilateral desaturation was characterized separately by interhemispheric rSO_2_ imbalance time and TCD flow asymmetry.

### 2.6 Postoperative delirium and cognitive outcomes

Postoperative delirium was the primary outcome. Richmond Agitation-Sedation Scale assessment was documented before each delirium assessment. Patients with RASS −4 or −5 were coded as unassessable at that time point and reassessed after sedation reduction or neurological improvement (Sessler et al., 2002). Delirium was identified from structured CAM-ICU records during ICU stay and standard CAM records after ICU discharge. Assessments were performed twice daily from postoperative day 1 to postoperative day 5, usually between 08:00–10:00 and 18:00–20:00. A positive CAM-ICU or CAM record at any assessment during postoperative days 1–5 defined postoperative delirium (Ely et al., 2001). Delirium duration was calculated as the number of postoperative days with at least one positive assessment. Delirium outcomes included incidence, assessability, and duration; CAM-S severity was not analyzed. Patients who remained unassessable because of coma, deep sedation, or early neurological deterioration were not coded as delirium in the primary analysis and were reclassified as unfavorable neurological outcomes in a sensitivity analysis.

Cognitive function was evaluated using the Chinese version of the Montreal Cognitive Assessment. Baseline MoCA was obtained within 7 days before elective surgery or within 24 h before urgent surgery when the patient was clinically assessable. Early postoperative MoCA was obtained on postoperative day 7 or before discharge when feasible. Follow-up MoCA was obtained at 30 ± 7 days through the outpatient follow-up clinic. One point was added for patients with ≤12 years of education according to the scale instruction (Nasreddine et al., 2005). The cognitive endpoint was MoCA-defined 30-day cognitive decline, defined as a decrease of at least 2 points from baseline. Patients with active delirium, stroke-related aphasia, severe hearing or visual impairment preventing assessment, or inability to complete MoCA at the 30-day visit were excluded from the observed-case cognitive-change analysis. This endpoint was analyzed as a pragmatic cognitive-function outcome, not as a formal diagnosis of postoperative neurocognitive disorder (Evered et al., 2018).

### 2.7 Biomarkers and clinical outcomes

Serum S100B, neuron-specific enolase, interleukin-6, and C-reactive protein were extracted from the laboratory information system. Routine sampling windows were before incision, 6 h after separation from cardiopulmonary bypass, and the morning of postoperative day 1. S100B, neuron-specific enolase, and interleukin-6 were measured using electrochemiluminescence immunoassays on the cobas e 801 platform. C-reactive protein was measured by immunoturbidimetry on the cobas c 702 analyzer. If more than one result was available within a sampling window, the value closest to the target time point was used.

Secondary clinical outcomes included ischemic stroke, transient ischemic attack, delayed awakening, mechanical ventilation duration, ICU length of stay, postoperative hospital length of stay, acute kidney injury, reoperation for bleeding, and in-hospital mortality. Stroke and transient ischemic attack were extracted from neurologist consultation notes, discharge diagnosis, and brain CT or MRI reports. Delayed awakening was defined as failure to regain purposeful response within 12 h after planned sedation discontinuation in the absence of documented deep hypothermia, residual neuromuscular blockade, or planned postoperative sedation. Mechanical ventilation duration was calculated from ICU admission to first successful extubation. ICU length of stay was calculated from ICU admission to ICU discharge, and postoperative hospital stay was calculated from surgery date to discharge or in-hospital death. Acute kidney injury was defined using KDIGO serum creatinine and urine-output criteria (Kidney Disease: Improving Global Outcomes (KDIGO) Acute Kidney Injury Work Group, 2012). As an exploratory secondary outcome, time of persistent organ dysfunction or death (TPOD) was calculated over the first 28 postoperative days as the cumulative time during which invasive mechanical ventilation, vasopressor/inotropic support, mechanical circulatory support, or renal replacement therapy was required; death was counted as persistent organ dysfunction through day 28 (Stoppe et al., 2016).

### 2.8 Data management and statistical analysis

Data were extracted from the electronic medical record, anesthesia information system, perfusion record, ICU nursing record, laboratory information system, operative report, discharge summary, and outpatient follow-up record. Patient identity was replaced with a coded study ID. Two trained reviewers independently checked demographic variables, operative variables, delirium records, cognitive records, and neurological outcomes. Disagreements were resolved by a senior investigator using the source record. Reviewers extracting postoperative delirium, cognitive outcome, and neurological outcome data were blinded to cohort assignment.

Signal segments were removed before analysis when they showed sensor detachment, absent signal-quality index, electrocautery artifact, probe displacement, nonphysiological jumps, or unstable values caused by documented technical interruption. Isolated missing periods shorter than 30 s were linearly interpolated when the values immediately before and after the gap were physiologically plausible. Longer artifact or missing segments were excluded. If a monitoring channel was unavailable for more than 20% of the relevant surgical phase, that channel was excluded from the derived burden calculation for that patient. Missing TCD data caused by absent transtemporal acoustic window were not replaced by normal values. Variables missing in at least 20% of patients were excluded from primary multivariable models and reported descriptively.

Continuous variables were summarized as mean ± standard deviation or median with interquartile range. Categorical variables were summarized as counts and percentages. Between-cohort comparisons used Student’s t test or Mann–Whitney U test for continuous variables and χ² test or Fisher’s exact test for categorical variables. Because cohort assignment was based on a time-defined protocol change, baseline imbalance and calendar-period confounding were addressed using propensity-score inverse probability weighting, multivariable regression, calendar-year adjustment, and prespecified sensitivity analyses. Because the exposure indicator was deterministically aligned with the January 1, 2024 protocol boundary, calendar-year adjustment was interpreted only as control for a gradual secular trend and not as proof that a contemporaneous level change from other causes had been excluded.

The propensity score included age, sex, body mass index, education years, hypertension, diabetes mellitus, prior stroke or transient ischemic attack, chronic kidney disease, left ventricular ejection fraction, emergency surgery, acute type A dissection, procedure type, and baseline MoCA. Calendar year was not included in the propensity-score model because cohort assignment was defined by protocol period; calendar year was instead included in the outcome regression models. Stabilized inverse probability weights were used, and extreme weights were truncated at the 1st and 99th percentiles. Covariate balance after weighting was considered acceptable when the standardized mean difference was <0.10. Propensity-score weighting procedures followed established balance diagnostics for observational studies (Austin and Stuart, 2015).

The primary model used weighted logistic regression for postoperative delirium, with cohort as the main exposure and robust standard errors. The covariate-adjusted model included age, baseline MoCA, emergency surgery, acute type A dissection, cardiopulmonary bypass time, circulatory arrest time, prior stroke or transient ischemic attack, and calendar year. Cerebral monitoring burden variables were not included in the primary model because they were downstream physiological variables affected by the guided protocol. A separate explanatory model added absolute rSO_2_ burden, EEG suppression burden, TCD asymmetry >30%, and COx >0.30 duration to examine attenuation of the cohort association after accounting for intraoperative cerebral pathophysiological burden.

MoCA-defined 30-day cognitive decline was analyzed using weighted logistic regression with the same core covariates plus postoperative delirium. Continuous 30-day MoCA change was analyzed using weighted linear regression. ICU length of stay and postoperative hospital length of stay were analyzed using generalized linear models when distributions were skewed. Missing covariate and 30-day cognitive data were handled using multiple imputation by chained equations with 20 imputed datasets when the variable-level missingness rate was below 20% (van Buuren and Groothuis-Oudshoorn, 2011). The imputation model included cohort, baseline covariates, operative variables, monitoring variables, delirium status, cognitive outcome, and clinical outcomes. Complete-case analysis was performed as a sensitivity analysis.

Additional sensitivity analyses excluded emergency surgery, excluded patients with postoperative stroke or transient ischemic attack, excluded the first 3 months after protocol implementation, restricted analysis to cases with complete NIRS and BIS records, restricted analysis to cases with complete NIRS, BIS, and TCD records, and reclassified patients never assessable for delirium as unfavorable neurological outcomes. Two-sided P <0.05 was considered statistically significant for the primary outcome. Secondary outcomes were interpreted as supportive. Biomarker analyses were adjusted using the Benjamini-Hochberg false-discovery-rate method. Analyses were performed using R version 4.4.3.

To examine secular trends around protocol implementation, an additional patient-level segmented logistic regression was fitted for postoperative delirium and MoCA-defined 30-day cognitive decline. The model included continuous time from study start, an indicator for the post-implementation period beginning January 1, 2024, and time elapsed after implementation. Time coefficients were scaled per 6 months. The three terms therefore estimated the pre-implementation slope, the immediate level change at implementation, and the change in slope after implementation, respectively. Descriptive semiannual outcome rates were plotted across January 2021–April 2026 with the implementation boundary marked at January 1, 2024.

## 3 Results

### 3.1 Cohort construction and baseline comparability

Between January 1, 2021 and April 30, 2026, 247 adult aortic arch surgery records were screened. After exclusion of 61 records, 186 patients were included in the final cohort: 92 in the conventional cerebral-protection cohort and 94 in the multimodal neuromonitoring-guided cohort. The main exclusion reasons were preoperative coma or documented dementia (n = 13), age >80 years (n = 9), death within 24 h after surgery (n = 7), incomplete anesthesia record (n = 7), and unavailable postoperative day 1–5 delirium documentation (n = 6). The full screening pathway is shown in **Supplementary Figure 1A**.

Primary delirium outcome ascertainment was complete for all 186 patients. Thirty-day MoCA follow-up was available in 173 patients, including 83 conventional-cohort patients and 90 guided-cohort patients. Thirteen patients lacked 30-day cognitive data because of in-hospital death, stroke-related aphasia, persistent delirium or inability to complete testing, or missed outpatient follow-up. During postoperative days 1–5, 32 patients had at least one unassessable delirium assessment, and 4 patients remained unassessable throughout the assessment window. These 4 patients were retained in the primary analysis and handled as unfavorable neurological outcomes in sensitivity analysis.

The two cohorts had comparable operative exposure. Cardiopulmonary bypass time was nearly identical between groups (202.9 ± 50.8 vs 203.1 ± 43.0 min), as were circulatory arrest time (32.5 ± 10.2 vs 32.1 ± 12.0 min), selective antegrade cerebral perfusion time (61.8 ± 16.7 vs 60.5 ± 17.4 min), and lowest nasopharyngeal temperature (25.5 ± 1.0 vs 25.4 ± 1.1 °C). Before weighting, the conventional cohort had lower education years (9.9 ± 3.2 vs 11.3 ± 3.0 years; P = 0.002), slightly lower baseline MoCA, and numerically higher proportions of emergency surgery and acute type A dissection. The guided cohort had more frequent conversion from unilateral to bilateral antegrade cerebral perfusion (46.2% vs 25.4%; P = 0.012) and shorter MAP <60 mmHg time (28.5 [14.0–48.0] vs 42.0 [25.0–61.0] min; P = 0.008). Most patients separated from cardiopulmonary bypass without major difficulty. Difficult separation occurred in 11/92 (12.0%) conventional-period patients and 9/94 (9.6%) guided-period patients (P = 0.600); temporary mechanical circulatory support at separation was required in 3/92 (3.3%) and 2/94 (2.1%), respectively (P = 0.680).

Inverse probability weighting reduced all prespecified baseline standardized mean differences to below 0.10. Calendar year was not included in the propensity-score model because cohort assignment was defined by protocol period; it was included in the outcome regression models. Covariate balance is shown in **Supplementary Figures 2A, B**, and baseline and operative characteristics are summarized in **Table 1**.

**Table 1:** Baseline and operative characteristics of the study cohort.

Variable	Conventional cohort n = 92	Multimodal-guided cohort n = 94	Unweighted P value	Weighted SMD
Age, years	62.9 ± 10.5	61.8 ± 10.4	0.470	0.04
Male sex	63/92 (68.5%)	52/94 (55.3%)	0.064	0.06
BMI, kg/m²	25.1 ± 3.4	25.3 ± 3.6	0.690	0.03
Education, years	9.9 ± 3.2	11.3 ± 3.0	0.002	0.07
Baseline MoCA	25.6 ± 1.7	26.1 ± 1.8	0.052	0.05
LVEF, %	59.8 ± 7.0	60.7 ± 7.5	0.390	0.04
EuroSCORE II, %	3.4 ± 1.3	3.2 ± 1.2	0.280	0.05
Hypertension	55/92 (59.8%)	62/94 (66.0%)	0.380	0.04
Diabetes mellitus	29/92 (31.5%)	23/94 (24.5%)	0.290	0.04
Chronic kidney disease	8/92 (8.7%)	12/94 (12.8%)	0.370	0.03
Prior stroke or TIA	8/92 (8.7%)	10/94 (10.6%)	0.650	0.03
Carotid stenosis ≥50%	5/92 (5.4%)	8/94 (8.5%)	0.410	0.05
Emergency surgery	52/92 (56.5%)	40/94 (42.6%)	0.057	0.06
Acute type A dissection	57/92 (62.0%)	50/94 (53.2%)	0.230	0.05
Chronic aortic dissection	20/92 (21.7%)	24/94 (25.5%)	0.540	0.04
Aortic arch aneurysm	15/92 (16.3%)	20/94 (21.3%)	0.380	0.05
Total arch replacement + FET	60/92 (65.2%)	54/94 (57.4%)	0.270	0.07
Total arch replacement without FET	10/92 (10.9%)	12/94 (12.8%)	0.690	0.03
Partial arch replacement	13/92 (14.1%)	15/94 (16.0%)	0.730	0.03
Other arch repair	9/92 (9.8%)	13/94 (13.8%)	0.390	0.05
Right axillary artery cannulation	54/92 (58.7%)	52/94 (55.3%)	0.640	0.04
Innominate artery cannulation	15/92 (16.3%)	17/94 (18.1%)	0.750	0.03
Direct arch-vessel cannulation	9/92 (9.8%)	8/94 (8.5%)	0.760	0.02
Combined cerebral-perfusion cannulation	14/92 (15.2%)	17/94 (18.1%)	0.600	0.04
Initial unilateral ACP	67/92 (72.8%)	65/94 (69.1%)	0.580	0.05
Initial bilateral ACP	25/92 (27.2%)	29/94 (30.9%)	0.580	0.05
Conversion from unilateral to bilateral ACP	17/67 (25.4%)	30/65 (46.2%)	0.012	0.08
Cardiopulmonary bypass time, min	202.9 ± 50.8	203.1 ± 43.0	0.980	0.03
Difficult separation from CPB	11/92 (12.0%)	9/94 (9.6%)	0.600	0.04
Temporary mechanical circulatory support at CPB separation	3/92 (3.3%)	2/94 (2.1%)	0.680	0.03
Aortic cross-clamp time, min	102.6 ± 32.8	99.8 ± 30.9	0.550	0.04
Circulatory arrest time, min	32.5 ± 10.2	32.1 ± 12.0	0.800	0.02
Selective ACP time, min	61.8 ± 16.7	60.5 ± 17.4	0.600	0.04
Lowest nasopharyngeal temperature, °C	25.5 ± 1.0	25.4 ± 1.1	0.640	0.03
Nadir hematocrit during CPB, %	24.2 ± 3.5	24.8 ± 3.3	0.230	0.05
Nadir hemoglobin during CPB, g/L	78.6 ± 12.1	80.4 ± 11.7	0.300	0.05
MAP <60 mmHg time, min	42.0 (25.0–61.0)	28.5 (14.0–48.0)	0.008	0.08
End-CPB lactate, mmol/L	3.4 (2.6–4.6)	3.2 (2.4–4.1)	0.210	0.05

Values are mean ± standard deviation, median (interquartile range), or n/N (%). ACP, antegrade cerebral perfusion; BMI, body mass index; CPB, cardiopulmonary bypass; FET, frozen elephant trunk; LVEF, left ventricular ejection fraction; MAP, mean arterial pressure; MoCA, Montreal Cognitive Assessment; SMD, standardized mean difference; TIA, transient ischemic attack.

### 3.2 Cerebral physiological burden during aortic arch surgery

The main intraoperative difference between cohorts was cerebral physiological burden rather than operative duration or cooling depth. NIRS data sufficient for burden calculation were available in 180 patients, BIS data in 174, and interpretable TCD data in 155. TCD availability was higher in the guided cohort than in the conventional cohort (91.5% vs 75.0%; P = 0.003), mainly because incomplete intraoperative TCD documentation was less frequent after protocol implementation. Missing TCD values were not treated as normal values. Phase-specific NIRS and BIS coverage remained high in both periods, with NIRS coverage ranging from 95.8% to 98.2% in the conventional cohort and 97.5% to 98.7% in the guided cohort, and BIS coverage ranging from 92.6% to 95.0% and from 95.2% to 96.8%, respectively.

Baseline rSO_2_ was almost identical between cohorts (66.6 ± 5.7% vs 66.7 ± 4.8%), but the guided cohort had a higher minimum rSO_2_ (48.8 ± 7.9% vs 44.8 ± 8.6%; P = 0.001). The oxygenation burden was substantially lower after protocol implementation: rSO_2_ <55% time decreased from 57.0 to 25.5 min, absolute rSO_2_ burden decreased from 123.8 to 41.1%-min, and relative rSO_2_ burden decreased from 174.2 to 82.5%-min, all with P < 0.001. Interhemispheric rSO_2_ imbalance time was also shorter in the guided cohort (15.0 vs 28.0 min; P = 0.002).

The same direction was observed for EEG suppression and autoregulation. EEG suppression burden was lower in the guided cohort (17.5 vs 21.5 min; P = 0.004), BIS <20 time was shorter (14.0 vs 24.0 min; P = 0.002), and COx >0.30 duration decreased from 34.0 to 21.0 min (P < 0.001). MAP outside the optimal range was also shorter in the guided cohort (38.0 vs 59.5 min; P < 0.001). In contrast, TCD-derived MCA velocity asymmetry and sudden MCA signal loss were directionally lower but did not reach statistical significance.

The reduction in physiological burden was accompanied by more protocol-driven corrective actions. The guided cohort had more MAP or ACP pressure interventions, PaCO_2_ correction, ACP flow or cannula adjustment, anesthetic reduction for EEG suppression, and rewarming slowdown. ACP flow or cannula adjustment was performed in 46.8% of guided patients compared with 22.8% of conventional patients (P < 0.001), while rewarming slowdown increased from 19.6% to 41.5% (P = 0.001). Transfusion for cerebral oxygenation did not differ significantly between cohorts. In the guided cohort, 128 NIRS-trigger episodes with a documented correction sequence occurred in 63 patients; 112 episodes (87.5%) were successfully corrected within 10 minutes. Because the conventional period did not use a fixed trigger-to-action pathway, an equivalent episode-level correction-success rate was not calculated for that cohort.

Biomarker results were supportive but not definitive. S100B on postoperative day 1 and IL-6 at 6 h and postoperative day 1 were lower in the guided cohort before adjustment, but these differences did not remain significant after false-discovery-rate correction. NSE and CRP showed no significant between-cohort differences. **Figures 1A–D** shows the rSO_2_ trajectory and distributions of rSO_2_ burden, EEG suppression burden, and impaired COx duration. Full intraoperative neuromonitoring, correction-action, and biomarker data are summarized in **Table 2**.

**Figure 1:**
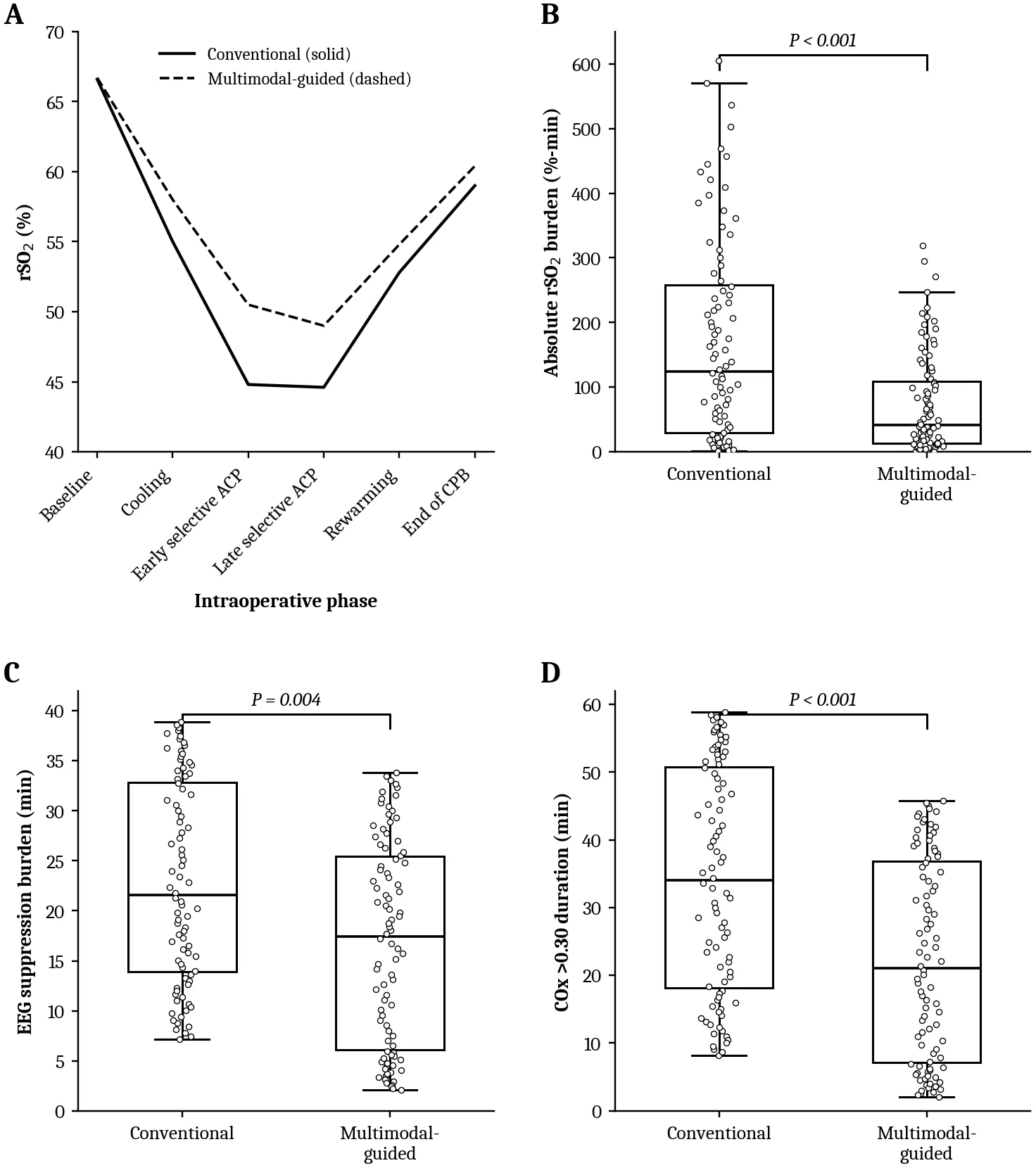
Intraoperative cerebral physiological burden. **(A)** Mean bilateral frontal rSO_2_ across baseline, cooling, early selective ACP, late selective ACP, rewarming, and end of CPB. **(B)** Absolute rSO_2_ burden by cohort. **(C)** EEG suppression burden by cohort. **(D)** Duration with COx >0.30 by cohort. For box plots in panels B–D, the center line denotes the median, the box the interquartile range, and whiskers extend to the most extreme observation within 1.5 × IQR; all individual observations are overlaid, including values beyond the whiskers. Thus, whiskers do not denote the observed sample maximum.

**Table 2:** Intraoperative neuromonitoring variables, correction actions, and biomarkers.

Variable	Conventional cohort	Multimodal-guided cohort	P value	FDR q value
NIRS data sufficient for burden calculation	88/92 (95.7%)	92/94 (97.9%)	0.440	—
BIS data sufficient for burden calculation	84/92 (91.3%)	90/94 (95.7%)	0.230	—
TCD data available	69/92 (75.0%)	86/94 (91.5%)	0.003	—
Absent transtemporal acoustic window	13/92 (14.1%)	6/94 (6.4%)	0.080	—
Incomplete TCD documentation	10/92 (10.9%)	2/94 (2.1%)	0.018	—
Baseline rSO_2_, %	66.6 ± 5.7	66.7 ± 4.8	0.890	—
Minimum rSO_2_, %	44.8 ± 8.6	48.8 ± 7.9	0.001	—
rSO_2_ <55% time, min	57.0 (26.8–87.0)	25.5 (0.0–54.5)	<0.001	—
Absolute rSO_2_ burden, %-min	123.8 (27.5–258.2)	41.1 (12.8–109.7)	<0.001	—
Relative rSO_2_ burden, %-min	174.2 (58.6–326.4)	82.5 (24.9–178.7)	<0.001	—
Interhemispheric rSO_2_ imbalance time, min	28.0 (12.0–45.0)	15.0 (5.0–31.0)	0.002	—
MCA velocity asymmetry, %	31.8 (18.8–41.1)	27.8 (17.2–39.7)	0.210	—
MCA velocity asymmetry >30%	33/69 (47.8%)	33/86 (38.4%)	0.240	—
Sudden MCA signal loss or marked reduction	9/69 (13.0%)	8/86 (9.3%)	0.470	—
EEG suppression burden, min	21.5 (13.8–33.0)	17.5 (6.0–25.5)	0.004	—
BIS <20 time, min	24.0 (12.0–36.0)	14.0 (5.0–26.0)	0.002	—
COx peak	0.40 ± 0.20	0.30 ± 0.20	0.004	—
COx >0.30 duration, min	34.0 (18.0–51.0)	21.0 (7.0–37.0)	<0.001	—
MAP outside optimal range, min	59.5 (41.5–78.0)	38.0 (15.0–53.8)	<0.001	—
Total documented correction actions, count	5.0 (3.0–6.0)	6.0 (4.2–8.0)	<0.001	—
NIRS trigger episodes with documented correction sequence	—	128 episodes in 63 patients	—	—
NIRS trigger episodes successfully corrected within 10 min	—	112/128 (87.5%)	—	—
MAP or ACP pressure intervention, count	3.0 (1.0–5.0)	5.0 (3.0–7.0)	<0.001	—
PaCO_2_ correction intervention, count	1.0 (0.0–2.0)	2.0 (1.0–3.0)	0.010	—
ACP flow or cannula adjustment	21/92 (22.8%)	44/94 (46.8%)	<0.001	—
Anesthetic reduction for EEG suppression	26/92 (28.3%)	43/94 (45.7%)	0.014	—
Rewarming slowdown	18/92 (19.6%)	39/94 (41.5%)	0.001	—
Transfusion for cerebral oxygenation	14/92 (15.2%)	21/94 (22.3%)	0.210	—
S100B before incision, μg/L	0.08 (0.06–0.12)	0.08 (0.06–0.11)	0.720	0.720
S100B 6 h after CPB, μg/L	1.04 (0.79–1.42)	0.96 (0.74–1.28)	0.120	0.240
S100B on POD1, μg/L	0.62 (0.41–0.89)	0.51 (0.34–0.77)	0.046	0.184
NSE before incision, ng/mL	13.2 ± 3.7	13.0 ± 3.5	0.700	0.720
NSE 6 h after CPB, ng/mL	25.1 ± 6.2	24.0 ± 5.8	0.210	0.336
NSE on POD1, ng/mL	22.6 ± 5.0	22.2 ± 5.0	0.580	0.663
IL-6 before incision, pg/mL	12.4 (7.2–20.8)	11.7 (6.9–19.6)	0.620	0.709
IL-6 6 h after CPB, pg/mL	392.0 (236.0–648.0)	318.0 (190.0–540.0)	0.048	0.184
IL-6 on POD1, pg/mL	468.0 (286.0–764.0)	356.0 (220.0–610.0)	0.031	0.184
CRP before incision, mg/L	4.8 (2.1–9.6)	5.0 (2.4–9.1)	0.810	0.810
CRP 6 h after CPB, mg/L	62.0 (40.0–91.0)	58.0 (36.0–86.0)	0.350	0.467
CRP on POD1, mg/L	108.0 (74.0–151.0)	96.0 (68.0–140.0)	0.190	0.336

Values are mean ± standard deviation, median (interquartile range), or n/N (%). FDR q values are shown for biomarker comparisons. ACP, antegrade cerebral perfusion; BIS, bispectral index; COx, cerebral oximetry index; CPB, cardiopulmonary bypass; CRP, C-reactive protein; FDR, false-discovery rate; IL-6, interleukin-6; MAP, mean arterial pressure; MCA, middle cerebral artery; NIRS, near-infrared spectroscopy; NSE, neuron-specific enolase; POD, postoperative day; rSO_2_, regional cerebral oxygen saturation; TCD, transcranial Doppler.

### 3.3 Postoperative delirium and cognitive outcomes

Postoperative delirium within postoperative days 1–5 occurred less frequently in the guided cohort than in the conventional cohort (14/94, 14.9% vs 36/92, 39.1%; P < 0.001). Delirium duration in the full cohort was shorter in the guided cohort, although duration among patients who developed delirium was similar between groups. Delayed awakening was less frequent in the guided cohort (9.6% vs 20.7%; P = 0.034), and mechanical ventilation duration was shorter (22.6 [13.8–41.2] h vs 29.2 [16.3–52.1] h; P = 0.048). Stroke or TIA, AKI, reoperation for bleeding, ICU stay, postoperative hospital stay, and in-hospital mortality did not differ significantly between cohorts. Exploratory TPOD was 31.0 (18.0–58.0) h in the conventional cohort and 24.0 (14.0–46.0) h in the guided cohort (P = 0.072).

The cognitive outcome followed the same direction. Among patients with available 30-day MoCA assessment, MoCA-defined 30-day cognitive decline occurred in 10 of 90 guided-cohort patients and 31 of 83 conventional-cohort patients (11.1% vs 37.3%; P < 0.001). Mean 30-day MoCA change was smaller in the guided cohort (−0.1 ± 1.6 points vs −1.2 ± 1.6 points; P < 0.001), and 30-day MoCA was higher after protocol implementation (25.9 ± 2.6 vs 24.4 ± 2.7; P < 0.001). **Figures 2A–D** presents delirium incidence, MoCA-defined 30-day cognitive decline, and the corresponding absolute rSO_2_-burden distributions stratified by clinical outcome status. Postoperative outcomes are summarized in **Table 3**.

**Figure 2:**
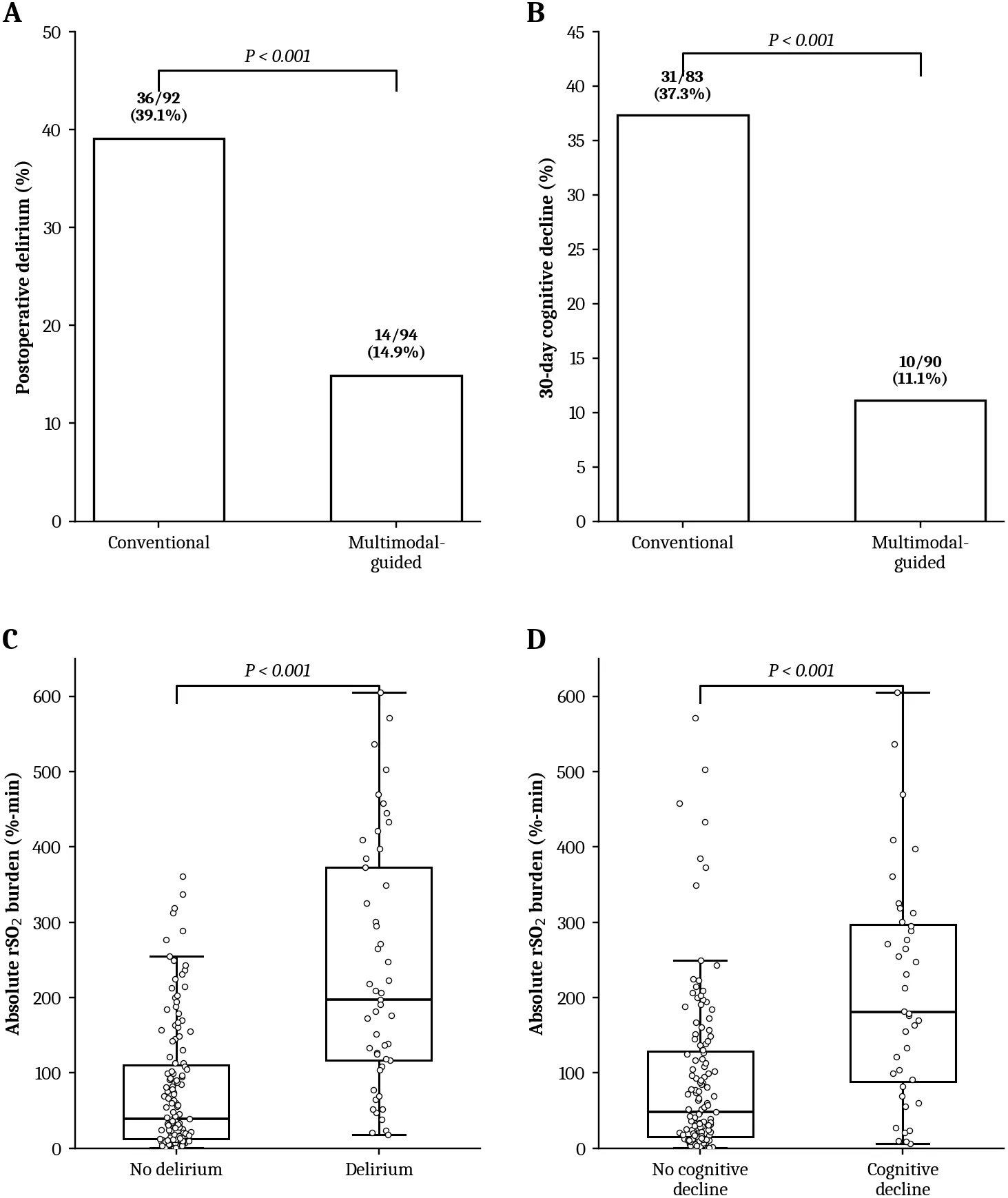
Postoperative outcomes and cerebral oxygenation burden. **(A)** Postoperative delirium incidence by cohort. **(B)** MoCA-defined 30-day cognitive decline by cohort. **(C)** Absolute rSO_2_ burden in patients with and without postoperative delirium. **(D)** Absolute rSO_2_ burden in patients with and without MoCA-defined 30-day cognitive decline. In panels C–D, box-plot conventions are identical to **Figures 1B–D**: center line, median; box, interquartile range; whiskers, most extreme observation within 1.5 × IQR; all individual observations are overlaid.

**Table 3:** Postoperative delirium, cognitive outcomes, and clinical outcomes.

Outcome	Conventional cohort	Multimodal-guided cohort	P value
At least one unassessable delirium assessment	19/92 (20.7%)	13/94 (13.8%)	0.210
Never assessable during POD1–5	3/92 (3.3%)	1/94 (1.1%)	0.360
Postoperative delirium within POD1–5	36/92 (39.1%)	14/94 (14.9%)	<0.001
Delirium duration, days	0.0 (0.0–2.0)	0.0 (0.0–0.0)	<0.001
Delirium duration among delirious patients, days	2.0 (1.0–3.0)	1.5 (1.0–3.0)	0.420
Delayed awakening	19/92 (20.7%)	9/94 (9.6%)	0.034
Ischemic stroke	2/92 (2.2%)	4/94 (4.3%)	0.680
Transient ischemic attack	1/92 (1.1%)	2/94 (2.1%)	1.000
Stroke or TIA composite	3/92 (3.3%)	6/94 (6.4%)	0.500
AKI, KDIGO stage ≥1	17/92 (18.5%)	19/94 (20.2%)	0.760
AKI, KDIGO stage ≥2	6/92 (6.5%)	7/94 (7.4%)	0.800
Reoperation for bleeding	6/92 (6.5%)	5/94 (5.3%)	0.720
Mechanical ventilation duration, h	29.2 (16.3–52.1)	22.6 (13.8–41.2)	0.048
Time of persistent organ dysfunction or death (TPOD), h	31.0 (18.0–58.0)	24.0 (14.0–46.0)	0.072
ICU length of stay, days	4.8 (3.6–7.2)	5.3 (3.5–6.7)	0.780
Postoperative hospital stay, days	23.0 (19.8–26.2)	21.0 (18.0–28.0)	0.140
In-hospital mortality	3/92 (3.3%)	0/94 (0.0%)	0.120
Baseline MoCA among 30-day follow-up patients	25.7 ± 1.7	26.1 ± 1.8	0.140
MoCA on POD7 or before discharge	23.9 ± 2.6	25.0 ± 2.5	0.004
MoCA at 30 days	24.4 ± 2.7	25.9 ± 2.6	<0.001
MoCA change at 30 days	−1.2 ± 1.6	−0.1 ± 1.6	<0.001
MoCA-defined 30-day cognitive decline	31/83 (37.3%)	10/90 (11.1%)	<0.001

Values are mean ± standard deviation, median (interquartile range), or n/N (%). AKI, acute kidney injury; ICU, intensive care unit; KDIGO, Kidney Disease: Improving Global Outcomes; MoCA, Montreal Cognitive Assessment; POD, postoperative day; TIA, transient ischemic attack.

Using the same patient-level absolute rSO_2_ burden variable and identical plotting convention in **Table 2**, and **Figures 1B** , **2C, D**, the outcome-stratified distributions remained within the same observed range. Median burden was 39.3%-min in patients without postoperative delirium and 196.8%-min in those with delirium (P < 0.001); among patients with 30-day cognitive follow-up and available rSO_2_ burden data, corresponding medians were 48.6 and 180.4%-min for patients without and with cognitive decline, respectively (P < 0.001).

### 3.4 Weighted outcome models and sensitivity analyses

In the primary weighted logistic regression model, the guided cohort was associated with lower odds of postoperative delirium after adjustment for age, baseline MoCA, emergency surgery, acute type A dissection, cardiopulmonary bypass time, circulatory arrest time, prior stroke or TIA, and calendar year (OR, 0.36; 95% CI, 0.17–0.77; P = 0.008). Older age, lower baseline MoCA, and longer cardiopulmonary bypass time were associated with higher delirium risk, whereas emergency surgery, acute type A dissection, circulatory arrest time, prior stroke or TIA, and calendar year were not independently associated with delirium in this model. The nonsignificant calendar-year coefficient was not interpreted as evidence against calendar-period confounding, because a continuous year term cannot separate the protocol indicator from another change occurring at the same implementation boundary.

After downstream cerebral physiological burden variables were added to the explanatory model, the guided-cohort estimate was attenuated (OR, 0.50; 95% CI, 0.22–1.15; P = 0.103). Absolute rSO_2_ burden, EEG suppression burden, and COx >0.30 duration remained associated with delirium, while TCD asymmetry >30% did not. For 30-day cognitive decline, the guided cohort remained associated with lower odds after weighting and adjustment (OR, 0.31; 95% CI, 0.13–0.75; P = 0.009), and postoperative delirium was associated with higher odds of cognitive decline (OR, 3.28; 95% CI, 1.31–8.20; P = 0.011).

The direction of the guided-cohort association was stable across sensitivity analyses. The association with lower delirium risk remained in complete-case analysis, after excluding emergency surgery, after excluding postoperative stroke or TIA, after excluding the first 3 months after protocol implementation, in patients with complete NIRS/BIS records, in patients with complete NIRS/BIS/TCD records, and when never-assessable patients were coded as unfavorable neurological outcomes. Sensitivity analyses for 30-day cognitive decline showed the same direction. Model estimates are listed in **Supplementary Table 2** and shown in **Supplementary Figures 3A–C**.

### 3.5 Segmented time-series analysis

In patient-level segmented logistic regression, the pre-implementation temporal slope was not significant for postoperative delirium (OR per 6 months, 1.06; 95% CI, 0.83–1.34; P = 0.663). At the January 2024 implementation boundary, the estimated odds decreased immediately (level-change OR, 0.20; 95% CI, 0.05–0.91; P = 0.037), with no evidence of an additional post-implementation slope change (slope-change OR per 6 months, 1.00; 95% CI, 0.61–1.63; P = 0.994). The cognitive endpoint showed a similar pattern: pre-implementation slope OR 1.04 (95% CI, 0.80–1.34; P = 0.783), immediate level-change OR 0.17 (95% CI, 0.03–0.92; P = 0.040), and post-implementation slope-change OR 1.00 (95% CI, 0.57–1.76; P = 0.987). Semiannual observed rates are shown in **Figure 3**.

**Figure 3:**
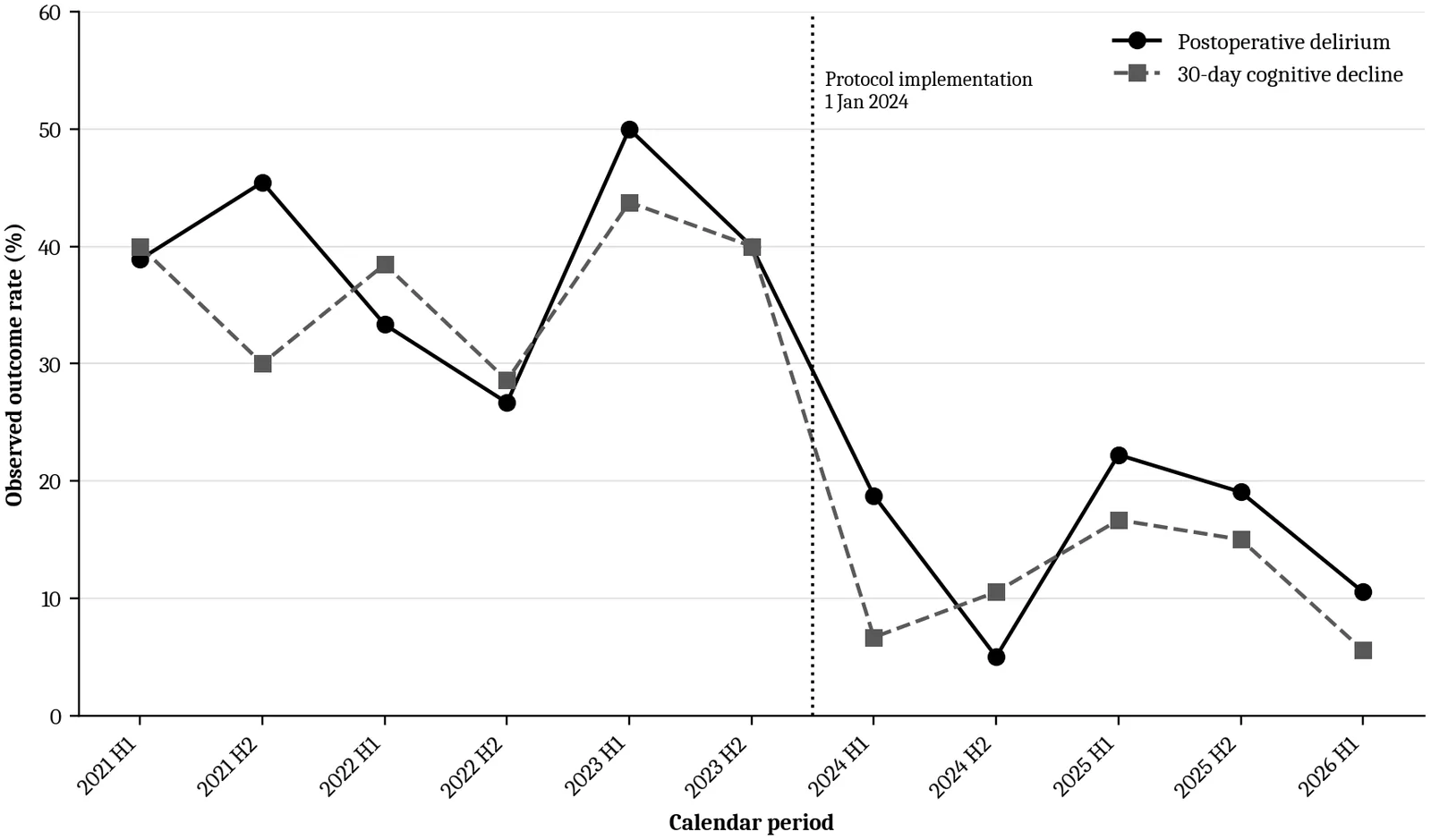
Descriptive semiannual trends in postoperative delirium and MoCA-defined 30-day cognitive decline from January 2021 to April 2026. The vertical dotted line marks implementation of the multimodal neuromonitoring-guided cerebral-protection protocol on January 1, 2024. The plotted rates are descriptive; inferential estimates are from the patient-level segmented logistic models.

## 4 Discussion

This retrospective cohort study examined a time-defined transition from conventional cerebral protection to multimodal neuromonitoring-guided cerebral protection in adult aortic arch surgery. The guided protocol was associated with lower intraoperative cerebral physiological burden and lower postoperative delirium risk. The segmented analysis did not identify a significant decline in delirium or 30-day cognitive decline before implementation; instead, both outcomes showed an estimated immediate level decrease at the January 2024 boundary without a further change in post-implementation slope. This temporal pattern is more compatible with a change occurring around implementation than with a steadily improving pre-existing secular trend, although it cannot exclude another co-intervention introduced at the same boundary. The attenuation after rSO_2_ burden, EEG suppression burden, and impaired COx duration were added to the explanatory model is consistent with a possible physiological pathway involving cerebral oxygenation, cortical suppression, and pressure autoregulation. The same direction was observed for MoCA-defined 30-day cognitive decline, whereas stroke, AKI, reoperation for bleeding, ICU stay, and postoperative hospital stay did not show parallel reductions.

Aortic arch surgery is a suitable setting for this strategy because neurological risk is produced by several overlapping mechanisms, including interrupted systemic circulation, selective antegrade cerebral perfusion, hypothermia and rewarming, arch-vessel manipulation, embolic load, anemia, carbon dioxide fluctuation, and variable autoregulation. Selective antegrade cerebral perfusion improves neurological safety, but it does not guarantee adequate bilateral cerebral flow during circulatory arrest (Spielvogel et al., 2013; Benedetto et al., 2022). Earlier studies using TCD and NIRS during antegrade selective cerebral perfusion showed that these modalities capture different aspects of cerebral perfusion and oxygenation during arch surgery (Harrington et al., 2007). In the present study, the guided cohort did not have shorter bypass or circulatory-arrest times, yet showed less desaturation burden, less EEG suppression burden, and shorter impaired COx duration. This finding supports the view that cerebral protection depends not only on the perfusion technique selected before arrest, but also on real-time correction of patient-specific physiological instability.

The results should not be interpreted as evidence that NIRS alone prevents postoperative delirium. A randomized trial in older cardiac-surgery patients found that cerebral oximetry-guided intervention did not significantly reduce delirium despite active correction of desaturation (Lei et al., 2017). Meta-analytic evidence is also mixed, with some analyses suggesting lower postoperative cognitive dysfunction but no consistent reduction in delirium or stroke across randomized trials (Serraino and Murphy, 2017). The present study differs from single-modality cerebral oximetry designs because NIRS was embedded in a protocol that also incorporated EEG suppression, TCD flow asymmetry, COx-guided pressure adjustment, PaCO_2_ correction, hemoglobin correction, anesthetic-depth adjustment, rewarming control, and selective ACP modification. In this framework, rSO_2_ burden was treated as a downstream physiological burden, not as a baseline confounder.

The EEG and COx findings strengthen the biological plausibility of the delirium result. Burst suppression and excessive cortical suppression during cardiac surgery have been associated with postoperative delirium, indicating that cerebral vulnerability is not captured by oxygenation alone (Pedemonte et al., 2020). NIRS-derived autoregulation monitoring can also identify pressure-passive cerebral oxygenation during cardiopulmonary bypass, so the same MAP may be adequate for one patient but inadequate for another (Hogue et al., 2021). In this study, rSO_2_ burden, EEG suppression burden, and impaired COx duration each carried outcome information in the explanatory model. TCD asymmetry was directionally consistent but not independently significant, probably because TCD availability was incomplete, acoustic windows varied between patients, and intermittent TCD documentation captured large-vessel flow asymmetry rather than the full perioperative brain-injury pathway.

The postoperative cognitive findings are consistent with the known relationship between delirium and early cognitive decline after cardiac surgery. Delirium after cardiac surgery has been associated with slower early cognitive recovery and lower cognitive performance after discharge (Saczynski et al., 2012). Other cardiac-surgery data also support an association between postoperative delirium and early cognitive decline, especially in the first postoperative month (Brown et al., 2018). Therefore, delirium and MoCA-defined 30-day cognitive decline should be viewed as related but distinct endpoints. Delirium reflects acute fluctuating brain dysfunction during the first postoperative days, whereas the 30-day MoCA endpoint reflects early recovery of global cognitive performance. The association between the guided protocol and both endpoints suggests that reducing intraoperative cerebral physiological burden may influence more than immediate emergence or ICU behavior. However, the MoCA endpoint remains a pragmatic cognitive outcome rather than a formal diagnosis of postoperative neurocognitive disorder.

The biomarker findings were less definitive than the physiological and clinical outcomes. S100B, NSE, IL-6, and CRP moved in the expected direction at some time points, but the differences did not remain robust after false-discovery-rate correction. This is clinically plausible because aortic arch surgery produces systemic inflammation, hemolysis, temperature shifts, bypass-related inflammatory activation, and variable tissue injury, which can dilute the specificity of circulating markers for brain injury. The absence of a strong biomarker signal prevents overinterpretation: the study supports an association between guided monitoring, lower cerebral physiological burden, and lower delirium or cognitive-decline risk, but it does not prove uniform prevention of neuronal injury.

Several limitations remain. First, cohort assignment was a deterministic function of protocol period rather than randomization. IPTW and covariate adjustment address measured imbalance, and the segmented analysis did not show a significant pre-implementation decline; nevertheless, an interrupted time-series model cannot separate the protocol from an unmeasured intervention introduced at the same January 2024 boundary. The period-specific review did not identify material changes in operator composition, maintenance anesthetic mix, dexmedetomidine or benzodiazepine exposure, early mobilization, formal ABCDEF-bundle implementation, or transfusion thresholds, but residual confounding from accumulated surgical, anesthesia, and perfusion experience remains possible. The observed increase in conversion from unilateral to bilateral ACP and reduction in MAP <60 mmHg time may reflect protocol-triggered actions, broader evolution of practice, or both. Second, TCD was intermittent rather than continuous, was less consistently available in the conventional period, and was not available in all patients; absence of a transtemporal acoustic window and differential documentation may therefore have introduced informative missingness and limited detection of transient flow abnormalities. Third, the absolute rSO_2_ burden definition used the higher simultaneous hemispheric value and therefore emphasized bilateral/global desaturation; isolated unilateral desaturation could be underrepresented by this burden metric and was interpreted together with interhemispheric rSO_2_ imbalance and TCD findings. Fourth, NIRS reflects regional frontal oxygenation and can be affected by extracranial tissue, sensor position, hemoglobin concentration, and device-specific assumptions; it should therefore be interpreted as a trend and burden measure rather than a direct whole-brain oxygenation measurement (Hogue et al., 2008). Fifth, postoperative cognition was assessed with MoCA rather than a full neuropsychological battery, limiting domain-specific interpretation. Sixth, the study was single-center, so the effect of the protocol may depend on local surgical technique, perfusion practice, anesthetic workflow, and staff familiarity with neuromonitoring. The monitoring protocol was intraoperative; routine NIRS, processed EEG, COx, and TCD monitoring was not continued after operating-room transfer, so delayed postoperative cerebral physiological disturbances could not be captured.

Overall, the findings support a practical interpretation: multimodal neuromonitoring is most useful when abnormal signals are linked to a prespecified correction pathway. Recording NIRS, EEG, TCD, or COx values alone is unlikely to change neurological outcomes. The clinically relevant step is the conversion of abnormal signals into timely correction of perfusion pressure, ACP flow, cannula position, PaCO_2_, hemoglobin, anesthetic depth, and rewarming speed. In adult aortic arch surgery, where neurological injury is multifactorial and phase-dependent, this integrated approach is more defensible than reliance on a single cerebral oxygenation threshold.

## 5 Conclusion

In adult patients undergoing open aortic arch surgery, implementation of a multimodal neuromonitoring-guided cerebral-protection protocol was associated with lower intraoperative cerebral oxygenation burden, less EEG suppression burden, shorter impaired autoregulation duration, lower postoperative delirium risk, and less MoCA-defined 30-day cognitive decline. These findings support the use of NIRS, processed EEG, TCD, and COx as complementary physiological signals within a structured correction pathway. Because this was a single-center retrospective cohort defined by a protocol-period transition, prospective multicenter studies are needed to determine whether this strategy causally improves postoperative neurocognitive outcomes.

## Data Availability

The original contributions presented in the study are included in the article/**Supplementary Material**. Further inquiries can be directed to the corresponding author.
