# Supplementary material for: Multimodal neuromonitoring-guided cerebral protection and early neurocognitive outcomes after aortic arch surgery
Source: Front Physiol. 2026 Sep 22;17:1948101. doi: 10.3389/fphys.2026.1948101 (PMC13638428; doi:10.3389/fphys.2026.1948101)
Supplement: Supplementary file 1 [file SupplementaryFile1.docx]

Supplementary Material

# Supplementary Figures and Tables

**Supplementary Figure S1.** Cohort construction and analytic workflow. (A) Patient screening and cohort allocation. (B) Perioperative data sources. (C) Signal processing and cerebral physiological burden derivation. (D) Delirium, cognitive, and clinical outcome assessment.

**Supplementary Figure S2.** Covariate balance before and after inverse probability weighting. (A) Absolute standardized mean differences before weighting. (B) Absolute standardized mean differences after weighting. The vertical line marks SMD = 0.10.

**Supplementary Figure S3.** Adjusted model estimates and sensitivity analyses. (A) Weighted logistic regression for postoperative delirium. (B) Weighted logistic regression for MoCA-defined 30-day cognitive decline. (C) Sensitivity analyses for the association between guided protocol implementation and postoperative delirium.

**Supplementary Figure S4.** Schematic intraoperative arrangement of multimodal neuromonitoring displays. Bilateral NIRS and processed EEG/BIS were displayed continuously, COx was derived from time-synchronized MAP and rSO₂ signals, and the TCD workstation was used for scheduled intermittent MCA flow-velocity assessments. Abnormal signals were reviewed by the anesthesia/perfusion team and linked to the structured correction pathway.

**Supplementary Table S1.** Prespecified neuromonitoring thresholds, derived variables, and correction actions.

| **Domain** | **Variable or threshold** | **Operational definition** | **Correction action in guided cohort** |
| --- | --- | --- | --- |
| Cerebral oxygenation | Baseline rSO₂ | Stable bilateral frontal rSO₂ after induction and before incision under FiO₂ 0.50, MAP 65–80 mmHg, and PaCO₂ 35–45 mmHg | Used as the reference for relative desaturation and relative burden |
| Cerebral oxygenation | rSO₂ <55% for ≥3 min | Either frontal hemisphere remained below 55% for ≥3 min | Check sensor, head position, arterial pressure, cannula position, ACP pressure, oxygenation, PaCO₂, hemoglobin, and flow |
| Cerebral oxygenation | rSO₂ decrease >20% from baseline for ≥3 min | Either hemisphere decreased to <80% of baseline for ≥3 min | Apply the same correction sequence as absolute desaturation |
| Cerebral oxygenation | Left–right rSO₂ difference >15% for ≥3 min | Interhemispheric difference exceeded 15 percentage points for ≥3 min | Reassess arch-vessel perfusion, cannula position, ACP pressure, and need for bilateral ACP |
| Oxygenation burden | Area below 55% | Minute-level sum of max{0, 55 − max[rSO₂,left, rSO₂,right]} across analyzable minutes; reported as %-min | Used as a downstream physiological exposure |
| Oxygenation burden | Area below 80% of baseline | Minute-level sum below 80% of patient-specific baseline rSO₂ | Used as a sensitivity exposure |
| Cerebral blood-flow velocity | MCA mean flow velocity | M1 MCA mean velocity measured at 45–55 mm depth | Used to confirm cerebral perfusion during cooling, selective ACP, rewarming, and post-bypass phases |
| TCD flow asymmetry | MCA velocity asymmetry >30% | Absolute left–right MCA mean velocity difference divided by the higher MCA velocity ×100% | Check probe position, cannula position, ACP pressure, and ACP flow; convert to bilateral ACP if persistent |
| Sudden MCA signal loss | Abrupt ipsilateral MCA signal loss or marked reduction | Flow loss after cannulation, arch-vessel clamping, or ACP initiation, excluding probe displacement | Immediate surgical and perfusion-team check |
| EEG suppression | BIS <20 or suppression ratio >20% at core temperature >34 °C | Excessive cortical suppression outside intended cooling or circulatory arrest | Reduce anesthetic depth when appropriate; reassess MAP, oxygenation, PaCO₂, temperature, glucose, and acid–base status |
| Cerebral autoregulation | COx >0.30 for ≥10 min | Moving correlation between 10-second MAP and rSO₂ over 5-minute windows remained >0.30 | Adjust MAP toward the range associated with lower COx and stable rSO₂ |
| Rewarming control | Blood–core temperature gradient ≥10 °C | Arterial outlet blood temperature exceeded core temperature by ≥10 °C | Slow rewarming and reassess NIRS, BIS, TCD, blood gas, and perfusion pressure |
| Hemoglobin correction | Hematocrit <24–26% with persistent desaturation | Desaturation persisted after correction of sensor, pressure, flow, oxygenation, and PaCO₂ | Red blood cell transfusion according to institutional protocol and bleeding status |
| Data quality | Monitoring channel unavailable for >20% of relevant phase | NIRS, BIS, or TCD missing, detached, unreadable, or artifact-contaminated for >20% of the phase used for that variable | Exclude that channel from burden calculation; do not replace missing TCD with normal values |

**Supplementary Table S2.** Weighted regression models and sensitivity analyses.

| **Model** | **Predictor or analysis** | **Effect estimate** | **95% CI** | **P value** |
| --- | --- | --- | --- | --- |
| Primary weighted model for POD | Guided cohort vs conventional cohort | OR 0.36 | 0.17–0.77 | 0.008 |
| Primary weighted model for POD | Age, per year | OR 1.05 | 1.01–1.10 | 0.015 |
| Primary weighted model for POD | Baseline MoCA, per point | OR 0.76 | 0.59–0.96 | 0.024 |
| Primary weighted model for POD | Emergency surgery | OR 1.32 | 0.60–2.93 | 0.490 |
| Primary weighted model for POD | Acute type A dissection | OR 1.19 | 0.52–2.70 | 0.680 |
| Primary weighted model for POD | CPB time, per 10 min | OR 1.08 | 1.01–1.16 | 0.031 |
| Primary weighted model for POD | Circulatory arrest time, per 5 min | OR 1.06 | 0.94–1.20 | 0.340 |
| Primary weighted model for POD | Prior stroke or TIA | OR 1.56 | 0.55–4.44 | 0.400 |
| Primary weighted model for POD | Calendar year | OR 0.94 | 0.72–1.21 | 0.610 |
| Explanatory POD model | Guided cohort vs conventional cohort | OR 0.50 | 0.22–1.15 | 0.103 |
| Explanatory POD model | Absolute rSO₂ burden, per 100 %-min | OR 1.20 | 1.03–1.40 | 0.020 |
| Explanatory POD model | EEG suppression burden, per 10 min | OR 1.13 | 1.01–1.29 | 0.047 |
| Explanatory POD model | COx >0.30 duration, per 10 min | OR 1.18 | 1.02–1.37 | 0.027 |
| Explanatory POD model | TCD asymmetry >30% | OR 1.35 | 0.67–2.71 | 0.400 |
| Weighted model for 30-day cognitive decline | Guided cohort vs conventional cohort | OR 0.31 | 0.13–0.75 | 0.009 |
| Weighted model for 30-day cognitive decline | Postoperative delirium | OR 3.28 | 1.31–8.20 | 0.011 |
| Weighted model for 30-day cognitive decline | Age, per year | OR 1.04 | 1.00–1.09 | 0.045 |
| Weighted model for 30-day cognitive decline | Baseline MoCA, per point | OR 0.88 | 0.68–1.13 | 0.310 |
| Sensitivity analysis for POD | Complete-case analysis | OR 0.39 | 0.18–0.86 | 0.019 |
| Sensitivity analysis for POD | Excluding emergency surgery | OR 0.34 | 0.12–0.96 | 0.041 |
| Sensitivity analysis for POD | Excluding postoperative stroke/TIA | OR 0.33 | 0.15–0.76 | 0.009 |
| Sensitivity analysis for POD | Excluding first 3 months after protocol implementation | OR 0.32 | 0.14–0.75 | 0.008 |
| Sensitivity analysis for POD | Complete NIRS and BIS records | OR 0.37 | 0.17–0.81 | 0.013 |
| Sensitivity analysis for POD | Complete NIRS, BIS, and TCD records | OR 0.41 | 0.18–0.95 | 0.038 |
| Sensitivity analysis for POD | Never-assessable patients coded as unfavorable neurological outcome | OR 0.40 | 0.19–0.83 | 0.014 |
| Sensitivity analysis for 30-day cognitive decline | Complete-case analysis | OR 0.34 | 0.14–0.83 | 0.018 |
| Sensitivity analysis for 30-day cognitive decline | Excluding postoperative stroke/TIA | OR 0.29 | 0.11–0.76 | 0.012 |
| Sensitivity analysis for 30-day cognitive decline | Excluding patients without POD7 MoCA | OR 0.33 | 0.13–0.84 | 0.020 |

BIS, bispectral index; CI, confidence interval; COx, cerebral oximetry index; CPB, cardiopulmonary bypass; MoCA, Montreal Cognitive Assessment; NIRS, near-infrared spectroscopy; OR, odds ratio; POD, postoperative delirium; rSO₂, regional cerebral oxygen saturation; TCD, transcranial Doppler; TIA, transient ischemic attack.

**Supplementary Table S3.** Team continuity, operator-volume summary, and major co-interventions by protocol period.

| **Characteristic** | **Conventional period** | **Multimodal-guided period** | **Summary** |
| --- | --- | --- | --- |
| Attending aortic surgeons | 3 core surgeons shared across both periods; 4 total study-wide | 3 core surgeons shared across both periods; 4 total study-wide | Stable core surgical team |
| Cardiac anesthesiologists | 4 shared across both periods; 6 total study-wide | 4 shared across both periods; 6 total study-wide | Stable core anesthesia team |
| Perfusionists | 4 shared across both periods; 5 total study-wide | 4 shared across both periods; 5 total study-wide | Stable core perfusion team |
| Center arch case volume | 43 (2021); 45 (2022); 46 (2023) | 47 (2024); 48 (2025); 18 (Jan–Apr 2026) | No abrupt center-volume change at implementation |
| Annual arch case volume per primary surgeon, complete calendar years | — | — | Median 11 cases/year (IQR, 9–14) across primary surgeons |
| Propofol maintenance anesthesia | 52/92 (56.5%) | 55/94 (58.5%) | Similar distribution |
| Sevoflurane maintenance anesthesia | 40/92 (43.5%) | 39/94 (41.5%) | Similar distribution |
| Postoperative dexmedetomidine exposure | 30/92 (32.6%) | 34/94 (36.2%) | No major period shift |
| Benzodiazepine exposure | 10/92 (10.9%) | 8/94 (8.5%) | No major period shift |
| 24-h intravenous morphine-equivalent opioid exposure, mg | 19.2 (12.6–27.8) | 18.5 (11.9–27.1) | Similar exposure |
| Early mobilization within 48 h | 54/92 (58.7%) | 61/94 (64.9%) | No protocol-boundary introduction |
| Formal ABCDEF-type bundle | No formal bundle introduced at the 2024 boundary | No formal bundle introduced at the 2024 boundary | Unchanged at implementation |
| CPB hematocrit target | ≥24% unless temporary clinical deviation was required | ≥24% unless temporary clinical deviation was required | Unchanged target |
| Transfusion approach | Institutional protocol based on oxygenation, hematocrit, and bleeding status | Institutional protocol based on oxygenation, hematocrit, and bleeding status | Unchanged approach |

Values are n/N (%) or median (interquartile range) unless otherwise indicated. Team-continuity counts describe personnel shared across both protocol periods. The available operator-volume summary is reported as the median annual number of arch cases per primary surgeon across complete calendar years.
